# Transposon mutagenesis in *Mycoplasma hyopneumoniae* using a novel mariner-based system for generating random mutations

**DOI:** 10.1186/1297-9716-44-124

**Published:** 2013-12-21

**Authors:** Gareth A Maglennon, Beth S Cook, Alannah S Deeney, Janine T Bossé, Sarah E Peters, Paul R Langford, Duncan J Maskell, Alexander W Tucker, Brendan W Wren, Andrew N Rycroft

**Affiliations:** 1 Department of Pathology and Pathogen Biology, The Royal Veterinary College, Hawkshead Lane, North Mymms, Hatfield, AL9 7TA, UK; 2 Section of Paediatrics, Imperial College London, St Mary’s Campus, London, W2 1PG, UK; 3 Department of Pathogen Molecular Biology, London School of Hygiene & Tropical Medicine, Keppel Street, London, WC1E 7HT, UK; 4 Department of Veterinary Medicine, University of Cambridge, Madingley Road, Cambridge, CB3 0ES, UK

## Abstract

*Mycoplasma hyopneumoniae* is the cause of enzootic pneumonia in pigs, a chronic respiratory disease associated with significant economic losses to swine producers worldwide. The molecular pathogenesis of infection is poorly understood due to the lack of genetic tools to allow manipulation of the organism and more generally for the *Mycoplasma* genus. The objective of this study was to develop a system for generating random transposon insertion mutants in *M. hyopneumoniae* that could prove a powerful tool in enabling the pathogenesis of infection to be unraveled. A novel delivery vector was constructed containing a hyperactive C9 mutant of the Himar1 transposase along with a mini transposon containing the tetracycline resistance cassette, *tetM. M. hyopneumoniae* strain 232 was electroporated with the construct and *tetM*-expressing transformants selected on agar containing tetracycline. Individual transformants contained single transposon insertions that were stable upon serial passages in broth medium. The insertion sites of 44 individual transformants were determined and confirmed disruption of several *M. hyopneumoniae* genes. A large pool of over 10 000 mutants was generated that should allow saturation of the *M. hyopneumoniae* strain 232 genome. This is the first time that transposon mutagenesis has been demonstrated in this important pathogen and could be generally applied for other Mycoplasma species that are intractable to genetic manipulation. The ability to generate random mutant libraries is a powerful tool in the further study of the pathogenesis of this important swine pathogen.

## Introduction

Belonging to the class *Mollicutes*, mycoplasmas are characterised by their lack of a cell wall and small genome size, and are considered to be the smallest free-living self-replicating organisms and as such are of considerable interest in synthetic biology [1]. Respiratory disease is a major problem facing swine producers and *Mycoplasma hyopneumoniae*, the cause of enzootic pneumonia (EP), is a swine-specific mycoplasma of global prevalence that is one of the leading causes of disease [2,3]. EP is characterised by a chronic non-productive cough that is most evident during the growing and fattening stages of production, although all ages of animal may be affected [4]. Mortality rates are usually low, but morbidity may be high with associated economic losses due to increased medication costs, lower growth rates and lower feed conversion efficiencies [2]. The pathogenesis of EP involves entry of *M. hyopneumoniae* into the respiratory tract by inhalation, largely from nose-to-nose contact with other pigs [2], and colonisation of the ciliated epithelial cells of the trachea, bronchi and bronchioles [5,6]. Adherence of the organism to the epithelium causes ciliostasis and loss of cilia, thereby preventing effective clearance of debris, pathogens and mucus from the airways [7]. Additionally, *M. hyopneumoniae* may cause direct cell damage by production of cytotoxic metabolites such as hydrogen peroxide [8]. The chronic nature of infection may result from modulation of the host immune response by *M. hyopneumoniae*[2,9] and possibly by variable expression of bacterial surface antigens, enabling the organism to evade effective clearance [10]. *M. hyopneumoniae* infection can be associated with and exacerbated by co-infection with other viral pathogens such as Porcine Reproductive and Respiratory Syndrome Virus (PRRSV) and Porcine Circovirus type 2 [2], and with upper respiratory tract bacteria such as *Actinobacillus pleuropneumoniae, Streptococcus suis, Haemophilus parasuis* and *Pasteurella multocida*[2]. Commercial *M. hyopneumoniae* vaccines are in widespread use in the pig industry where they are reported to lessen the economic effects of the disease by reducing clinical signs and lung lesions and by improving performance parameters [3,11,12]. However, current vaccines are not completely effective and do not prevent colonisation of the respiratory tract with *M. hyopneumoniae* or eliminate infection from the herd [13].

Central to the design of more effective vaccines is an understanding of the pathogenesis of EP. Despite the availability of whole genome sequences of several *M. hyopneumoniae* strains and the small genome size, the functions of most of the protein coding sequences are still unclear. The chronic nature of *M. hyopneumoniae* infection implies a complicated relationship between the pathogen and the host that is poorly understood. The small genome size of *M. hyopneumoniae* offers an appealing opportunity to understand the molecular basis of disease formation, and to unravel the interplay between the host and the pathogen. Traditionally the exploration of gene function in a bacterium is investigated through the generation of random insertional mutants by transposon mutagenesis. Typically for other Mycoplasmas, the Tn*4001* transposon derived from *Staphylococcus aureus*[14] has been utilised [15-19]. As reviewed by Halbedel and Stülke [20], a mini Tn*4001* transposon is used containing an antimicrobial resistance cassette flanked by inverted repeat sequences. The mini transposon and transposase enzyme are delivered into the organism by transformation and expression of the resistance cassette enables selection of mutated organisms. Insertions in essential genes result in lethality but many transformants have mutations in genes that are not necessary for growth in vitro, but may encode for particular pathogenicity determinants that are necessary for growth, survival, invasion or disease in the animal. Such mutants could be exploited in a number of different assays, including in vivo screening of mutants using the powerful technique of signature tagged mutagenesis [21]. Through the screening of large numbers of random mutants in vivo, mutants that are attenuated in vivo can be identified. Such mutants may encode for virulence factors and may be useful in the identification of live attenuated vaccine candidates.

Recently we described the first successful transformation of *M. hyopneumoniae* strain 232 using an artificial self-replicating *oriC* plasmid system [22]. This system allowed us to optimise a set of transformation conditions, antimicrobial selection cassettes and promoter sequences for *M. hyopneumoniae*. In this study, the results of that work have been used further to develop a transposon mutagenesis system for *M. hyopneumoniae*. A key feature of this system is the use of a Himar1 transposon, belonging to the Mariner family of transposons. Insertion occurs at any TA dinucleotide [23] offering an excellent potential for complete coverage of the AT-rich genome of *M. hyopneumoniae*. We describe the development of this system and its use in generating a large pool of several thousand *M. hyopneumoniae* mutants. This is a major advancement in the study of this important swine pathogen and potentially other Mycoplasmas.

## Materials and methods

### Plasmid construction

**Figure 1:**
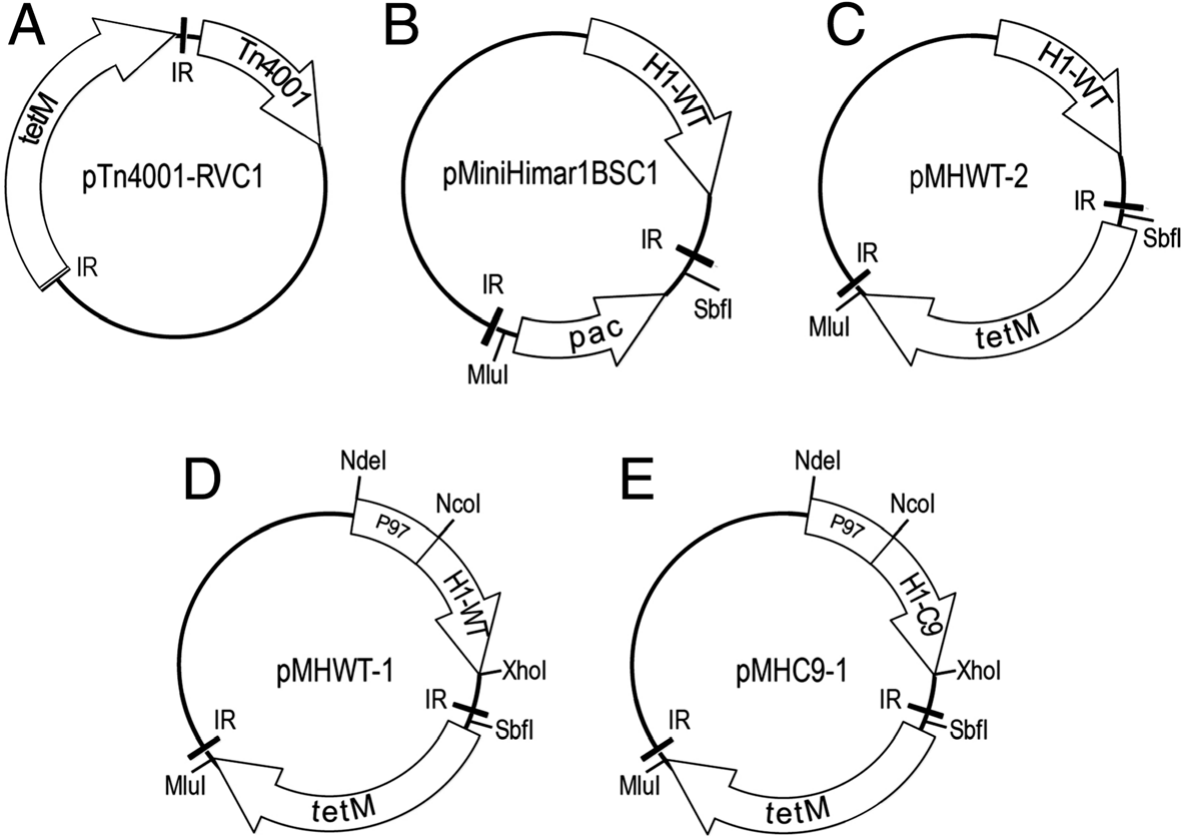
**Plasmid maps and construction.** Plasmid pTn4001-RVC1 was constructed by replacing the *pac* gene of pMiniTn4001PsPuro with *tetM* from plasmid pSRT2 **(A)**. Plasmid pMiniHimar1BSC1 contains a mini transposon incorporating *pac* conferring resistance to puromycin and the wild-type Himar1 transposase downstream of the Tn*4001* native promoter sequence **(B)**[26]. In the pMHWT-2 plasmid, the *pac* in pMiniHimar1BSC1 has been replaced with *tetM* from pSRT2 **(C)**. In plasmid pMHWT-1, the promoter sequence of the *M. hyopneumoniae* strain 232 P97 gene has been placed upstream of the wild-type Himar1 gene **(D)**. In pMHC9-1, the wild-type Himar1 gene has been replaced with the hyperactive C9 mutant version of the gene **(E)**[28].

PCR was performed using a Phusion High Fidelity kit (NEB Ltd, Hitchin, UK) according to the manufacturer’s instructions. Sequences of oligonucleotides used in the study are shown in Table 1. To construct pTn4001-RVC1 (Figure 1A), the 2293 base pair (bp) *tetM* gene of the plasmid pSRT2 [24] was amplified by PCR (primers TetM1 and TetM2) and cloned into the *Xba*I and *Pst*I restriction sites of the pMiniTn*4001*PsPuro plasmid [25]. Mariner transposon plasmids were constructed from the plasmid pMiniHimar1BSC1 (Figure 1B) [26]. To construct pMHWT-2, firstly the 2293 bp *tetM* gene in pSRT2 [24] was amplified by PCR (primers TetM3 and TetM4) and used to replace the puromycin N-acetyl-transferase (*pac*) gene of pMiniHimar1BSC1 using the *Mlu*I and *Sbf*I restriction sites (Figure 1C). pMiniHimar1BSC1 contains the Himar1 transposase gene downstream of the native promoter sequence from the Tn*4001* transposase. To produce plasmid pMHWT-1 (Figure 1D), the 1050 bp Himar1 transposase gene minus the Tn*4001* promoter sequence was PCR-amplified from pMiniHimar1BSC1 (primers Tpn1 and Tpn2), introducing an *Xho*I cut site at the 3’ end and *Nde*I and *Nco*I restriction sites at the 5’ end. This PCR product was cloned into *Nde*I and *Xho*I sites of pMHWT-2, replacing the Himar1 gene and Tn*4001* promoter sequence, and introducing a new *Nco*I cloning site. Next, the 619 bp P97 ciliary adhesin gene promoter sequence was amplified from *M. hyopneumoniae* strain *232*[27] genomic DNA with *Nco*I and *Nde*I restriction sites added (primers P97-1 and P97-2). The P97 promoter sequence was then cloned into the *Nco*I and *Nde*I restriction sites upstream of the Himar1 transposase gene, placing Himar1 under its control, and completing plasmid pMHWT-1. To generate plasmid pMHC9-1 (Figure 1E), the 1050 bp hyperactive C9 transposase mutant of *Himar1* was PCR amplified from plasmid pET29b + C9 [28], adding *Xho*I and *Nco*I restriction sites (primers Tpn1 and Tpn2). This PCR product was used to replace the wild-type Himar1 transposase gene of pMHWT-1, generating plasmid pMHC9-1 [GenBank accession number KF861545] such that the C9 mutant Himar1 is under control of the P97 promoter sequence.

**Table 1:** Sequences of oligonucleotides.

**Oligonucleotide**	**Restriction sites**	**Oligonucleotide sequence (5’-3’)**
TetM1	*Pst*I	GCCGCTGCAGAATTAAAAGTTAGTG
TetM2	*Xba*I	GAAATCTAGATTATATAACAACTTAAATTAC
TetM3	*Sbf*I	CGATCCTGCAGGCAGAATTAAAAGTTAGTG
TetM4	*Mlu*I	CAGTACGCGTTTATATAACAACTTAAATTAC
Tpn1	*Nde*I/NcoI	GCGCATATGCCATGGAAAAAAAGGAATTTCGTG
Tpn2	*Xho*I	CGCCACTCGAGATTATTCAACATAGTTCCCTTC
P97-1	*Nde*I	CGATCATATGACGGGGATTTAAAACAGAAAC
P97-2	*Nco*I	GAATCCATGGCACCAACAATTCCGGCAGTC
TetMF	N/A	GTGGACAAAGGTACAACGAG
TetMR	N/A	CGGTAAAGTTCGTCACACAC
Linker-A	N/A	CGACTGGACCTGGA
Linker-B	N/A	GATAAGCAGGGATCGGAACCTCCAGGTCCAGTCG
L-PCR-C	N/A	GATAAGCAGGGATCGGAACC
L-PCR-L	N/A	GATAGGGTTGAGTGTTGTTC
L-PCR-R	N/A	TAGTCGGATAGATAAAGTAC

Sequences of oligonucleotides used in the study are shown. Where appropriate, the restriction enzyme recognition sites added to the 5’ end of the oligonucleotides are underlined.

### Bacterial culture

*M. hyopneumoniae* strain 232 [27] was grown in Friis broth medium at 37 °C in a static incubator [29]. For growth on solid medium, Friis medium was solidified by addition of 0.8% w/v purified agar (Oxoid Ltd, Basingstoke, UK) and 0.01% w/v DEAE-dextran (Sigma-Aldrich Ltd, Gillingham, UK) and incubated at 37 °C with 5% CO_2_. For the selection of transformants, tetracycline hydrochloride (Sigma-Aldrich Ltd, Gillingham, UK) was added to Friis broth medium and Friis agar medium at final concentrations of 0.5 μg/mL and 0.2 μg/mL respectively. Molecular cloning was performed using *Escherichia coli* strain DH5α, grown in Luria-Bertani medium according to standard methods [30]. Transformants were selected by the addition of 100 μg/mL ampicillin (Sigma-Aldrich Ltd, Gillingham, UK) to the medium. Additionally, for the selection of transformants containing the *tetM* gene, tetracycline hydrochloride was added to a final concentration of 5 μg/mL.

### Transformation of mycoplasmas

*M. hyopneumoniae* strain 232 was grown to mid-late logarithmic phase in Friis broth as determined by an acid colour change in the phenol red pH indicator. Mycoplasmas were harvested by centrifugation of culture at 9000 × g for 10 min at 4 °C and washed three times in electroporation buffer (272 mM sucrose, 8 mM HEPES, pH 7.4). One hundred microliters of cells (corresponding to 3 mL culture) were incubated on ice with approximately 10 μg plasmid DNA for 30 min. Electroporation was performed in a 0.2 cm cuvette (Bio-Rad Ltd, Hemel Hempstead, UK) at 2.5 kV, 100 Ω, 25 μF and 900 μL ice-cold Friis medium were immediately added. After 15 min incubation on ice, cells were transferred to a 1.5 mL tube and incubated at 37 °C for 3 h. Culture was then plated onto Friis agar containing tetracycline and incubated at 37 °C in 5% CO_2_ for up to 18 days. Individual tetracycline-resistant transformants were picked using a sterile pipette tip into Friis broth medium containing tetracycline and grown for 5–7 days until evidence of growth as determined by the pH indicator.

### Determination of transposon insertion sites

Transposon insertion site determination was based on the method described by Chaudhuri et al. [31]. Genomic DNA was extracted from *M. hyopneumoniae* strain 232 culture using a phenol-chloroform method [32]. Presence of *tetM* and plasmid backbone was determined by PCR using primer pairs TetMF/TetMR and Tpn1/Tpn2 respectively. Two and a half micrograms of DNA were digested overnight at 37 °C with 10 units of *Alu*I, which cuts the 892 kbp genome of *M. hyopneumoniae* strain 232 a total of 2239 times and generates blunt ends. *Alu*I also cuts within the *tetM* transposon sequence six times. The digested DNA was purified using a MinElute PCR Purification kit (Qiagen Ltd, Manchester, UK). Oligonucleotide linkers were attached to the digested DNA fragments. To generate the linker, 10 μM oligonucleotides Linker-A and Linker-B (Table 1) were heated together in a boiling water bath for 3 min in annealing buffer (10 mM Tris–HCl, 50 mM NaCl, 1 mM EDTA, pH 8.0) and then allowed to cool for 1 h at room temperature. Fifty nanograms of *Alu*I digested DNA were blunt-ligated to 4 μL annealed oligonucleotides Linker-A and Linker-B in a 10 μL reaction using a Quick Ligation kit (NEB Ltd, Hitchin, UK) for 1 h at room temperature. The ligated DNA was purified using a MinElute PCR Purification kit (Qiagen Ltd, Manchester, UK). PCR was performed using HotStarTaq (Qiagen Ltd, Manchester, UK) according to the manufacturer’s instructions. Thermocycler conditions were as follows: 95 °C for 15 min; 33 cycles of 94 °C for 45 s, 55 °C for 60 s, 72 °C for 120 s; 72 °C for 10 min. PCR products were visualised by electrophoresis in 1.6% agarose. Transposon insertion sites were determined by the direct sequencing of PCR products.

### Southern analysis

Southern blotting was performed based on previously described methods [22]. Total DNA was extracted from 20 mL mycoplasma broth culture using a phenol-chloroform method [30,32] and 2.5 μg were digested to completion with *Hin*dIII. DNA was separated by electrophoresis on 0.9% agarose, blotted onto Hybond-N + membrane (GE Healthcare Ltd, Little Chalfont, UK) and then fixed to the membrane by exposure to UV light. A digoxigenin (DIG)-labeled probe specific for the *tetM* gene was generated from PCR-amplified DNA (primers TetMF and TetMR, Table 1) using a DIG-High Prime DNA Labelling and Detection Starter Kit II (Roche Applied Science Ltd, Burgess Hill, UK). This kit was also used to perform pre-hybridisation and hybridisation in accordance with the manufacturer’s instructions. The membrane was autoradiographed at room temperature using CL-XPosure Film (Fisher Scientific Ltd, Loughborough, UK).

## Results

### Construction of transposon delivery vectors

Plasmid pTn4001-RVC1 (Figure 1A) was constructed containing the Tn*4001* transposase and a mini Tn*4001* transposon consisting of the *tetM* gene under control of the spiralin gene promoter sequence of *Spiroplasma citri* bounded by inverted repeats. We previously optimised a set of transformation conditions for *M. hyopneumoniae* using an *oriC*-based self-replicating plasmid system, and showed that *tetM* under control of the spiralin gene promoter region was successfully expressed in *M. hyopneumoniae* strain 232 allowing the selection of transformants on Friis agar plates containing 0.2 μg/mL tetracycline [22]. However, despite three separate attempts at transforming *M. hyopneumoniae* strain 232 with pTn4001-RVC using our optimised conditions and our *oriC* plasmid pMHO-2 [22] as a positive control for transformation, we obtained no transformants. This suggested that the Tn*4001* transposase was not functional in *M. hyopneumoniae*.

**Figure 2:**
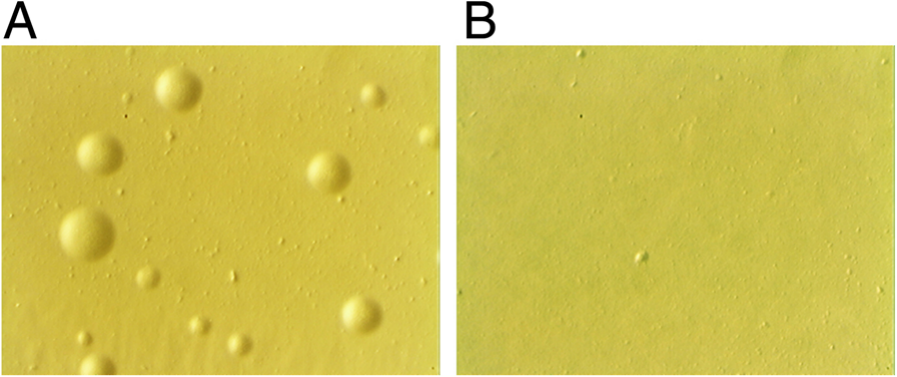
**Microscopic appearance of mutant *M. hyopneumoniae *colonies. ***M. hyopneumoniae* strain 232 was transformed with plasmid pMHC9-1 and within 14 days, tetracycline resistant colonies formed on Friis agar **(A)**. No colonies were observed on “no DNA” control transformations **(B)**. Individual colonies exhibited a similar appearance to non-transformed *M. hyopneumoniae* colonies. Tiny deposits on the surface of the agar are typically seen and are considered to arise from dead *M. hyopneumoniae* that have failed to grow in the presence of tetracycline or from proteinaceous material in the fresh yeast extract used **(A and B)**.

A Himar1 transposon delivery vector (pMiniHimar1BSC1) was recently described that successfully generated transposon insertion mutants in the *M. gallisepticum* R_(low)_ strain and a small number of transposon insertions in *M. hyopneumoniae* strain 232 [26]. pMiniHimar1BSC1 consists of the Himar1 transposase under control of the promoter sequence from Tn*4001* and a transposon carrying the *pac* gene conferring resistance to puromycin (Figure 1B). Small numbers of mutants (less than 10 per transformation) were generated in *M. hyopneumoniae* using this vector, but transformation frequencies were very low and protracted recovery times (several days) were required after transformation prior to selection of transformants on solid medium. We attempted to transform *M. hyopneumoniae* with pMiniHimar1BSC1 and using a self-replicating *oriC* plasmid (pMHOpuro) as a positive control for transformation [22]. Approximately 10^6^ CFU were transformed with pMiniHimar1BSC1 and with pMHOpuro. On this occasion, no puromycin resistant transformants were generated with plasmid pMiniHimar1BSC1 while an average of 1.5 × 10^-4^ transformants/CFU were produced by the pMHOpuro positive control. To determine whether *tetM* selection would increase the yield of transformants, *pac* was replaced with *tetM* in plasmid pMHWT-2 (Figure 1C). An alternative hypothesis for the paucity of transformants was that the Tn*4001* promoter sequence controlling Himar1 transposase expression was poorly active in *M. hyopneumoniae* hence pMHWT-1 (Figure 1D) was made by replacing the Tn*4001* promoter sequence regulating Himar1 with the P97 gene promoter of *M. hyopneumoniae* strain 232 [22]. Plasmid pMHC9-1 (Figure 1E) was constructed by replacing the wild-type Himar1 transposase with the C9 mutant form which exhibits increased activity in vitro [28]. Additionally, as a positive control for transformation, mycoplasmas were transformed with the *oriC*-based plasmid pMHO-2 (containing *tetM*). Approximately 10^6^ CFU were transformed with pMHWT-2, pMHWT-1, pMHC9-1 and pMHO-2 and selection plates examined after incubation for 14 days. Plasmid pMHWT-2 failed to generate any tetracycline resistant colonies compared to an average of 4.6 × 10^-5^ transformants/CFU for the pMHO-2 positive control Table 2. An average of 123 colonies per transformation were found for pMHC9-1 containing the hyperactive C9 mutant transposase compared to 33 colonies for pMHWT-1 containing the wild-type transposase. Transformation was repeated using pMHWT-1 and pMHC9-1 but with approximately 10^8^ CFU per transformation. Again, more transformants were generated with the hyperactive transposase (33 per transformation) compared to the wild-type transposase (10 per transformation). Transformation of an increased number of cells resulted in a drop in the overall transformation efficiency. However, our experiments suggested that the hyperactive C9 mutant Himar1 transposase could account for a 3-4-fold increase in transformation efficiency compared to the wild-type transposase. Morphologically, tetracycline-resistant colonies grown on Friis agar from transformed cells had a uniform appearance that resembled that of colonies grown from non-transformed cells (Figure 2). However, transformants took approximately 3–5 days longer to reach the same colony size as non-transformed cells. Transformed cells formed discrete and well-separated colonies allowing the isolation of individual colonies. Additionally, colonies were relatively non-adherent to the agar surface and could be easily removed by gentle washing with Friis medium. Importantly, in none of the experiments described did we observe colonies in our “no DNA” control transformations, indicating the absence of pseudo-resistance or spontaneous resistance to tetracycline.

**Table 2:** Transformation frequencies using Himar1 constructs.

**Plasmid**	** Mean transformation frequency (transformants/CFU) [SE]**	
	**Experiment 1**	**Experiment 2**
pMHWT-2	0	NT
pMHWT-1	2.5 × 10^-6^ [1.3 × 10^-6^]	9.0 × 10^-8^ [2.4 × 10^-8^]
pMHC9-1	9.3 × 10^-6^ [4.5 × 10^-6^]	3.0 × 10^-7^ [4.6 × 10^-8^]

Mean transformation frequencies (with standard errors) are shown for three separate transformations of *M. hyopneumoniae* strain 232 using plasmid constructs pMHWT-1, pMHWT-2 and pMHC9-1 in two separate experiments. In experiment 1, 10^6^ CFU were transformed with each plasmid while in experiment 2, 10^8^ CFU were transformed.

### Analysis of transformants

**Figure 3:**
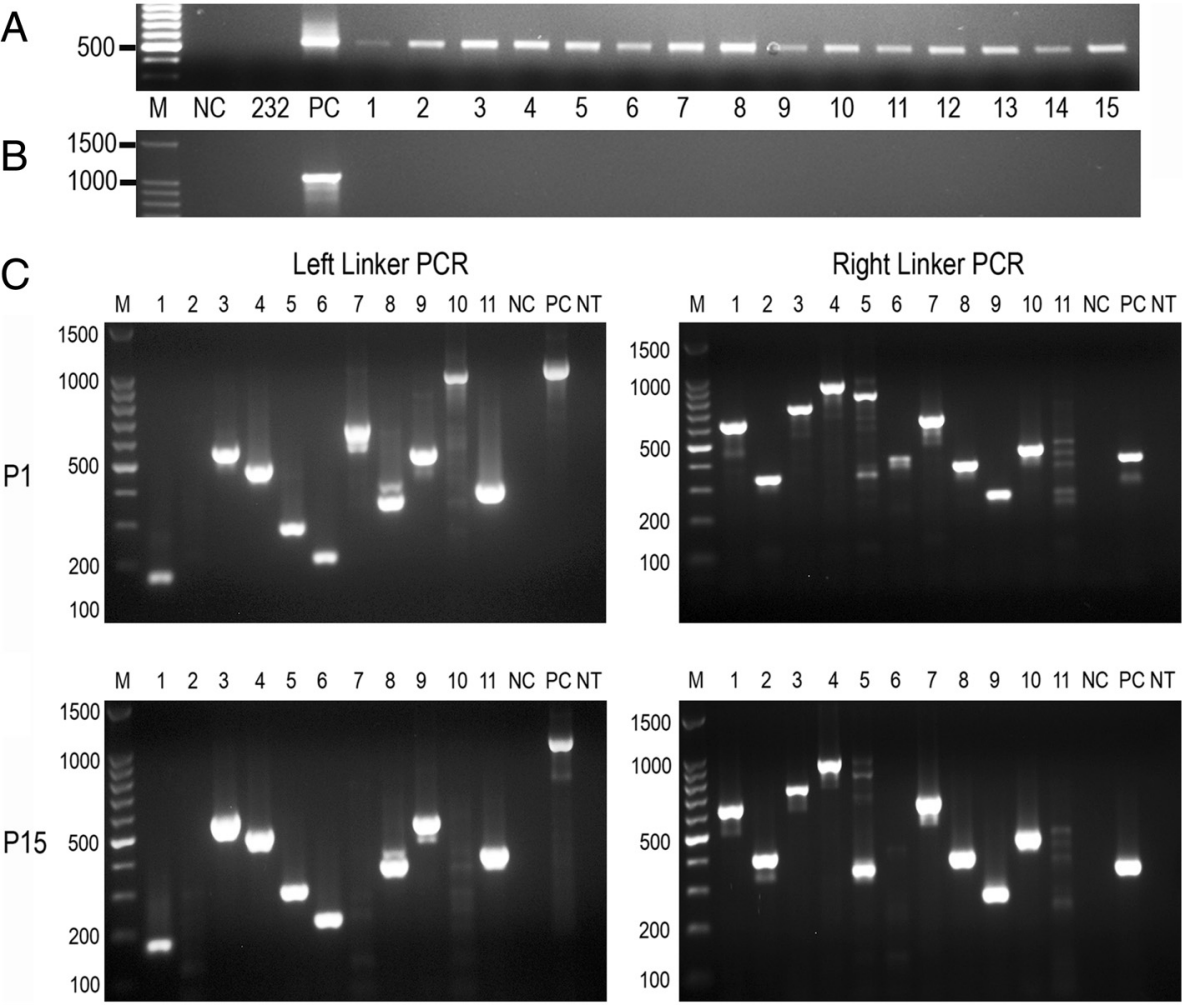
**Analysis of mutant *M. hyopneumoniae *by PCR. ***M. hyopneumoniae* strain 232 was transformed with pMHC9-1. PCR was performed on extracted total DNA from 15 individual transformants (lanes 1–15) for identification of the tetM gene **(A)** and the pGEM-T plasmid backbone **(B)**. A further 11 transformants underwent a further two rounds of “colony purification”. Total DNA was extracted and linker PCR performed using two primer pairs at either end of the transposon (left and right) **(C)**. The same 11 transformants were passaged 15 times in Friis medium without selection, and all subsequently retained their resistance to tetracycline. Linker PCR was repeated using the same two primer pairs (left and right). Control samples for PCR reactions included: “no template” control (NT); untransformed *M. hyopneumoniae* strain 232 (NC); plasmid DNA positive control (PC).

Transformation of *M. hyopneumoniae* strain 232 with plasmid pMHC9-1 consistently generated tetracycline resistant colonies on Friis agar containing tetracycline. To confirm that tetracycline resistance was due to transposition into the host chromosome and not, for example, due to maintenance of extrachromosomal plasmid DNA, 15 individual colonies from pMHC9-1 transformed *M. hyopneumoniae* strain 232 were analysed. Each was transferred into 1 mL Friis broth medium containing 0.5 μg/mL tetracycline. All transformants grew within 5 days, confirming the maintenance of tetracycline resistance upon sub-culturing into liquid medium while no growth was observed for untransformed controls. PCR amplification of *tetM* from total DNA extracts prepared from 20 mL culture confirmed the presence of *tetM* in all transformants generated with the hyperactive C9 mutant transposase but not un-transformed *M. hyopneumoniae* strain 232 (Figure 3A). In addition, PCR for a region of the pUC19 plasmid backbone failed to amplify DNA for any transformant showing that tetracycline resistance was not due to maintenance of extrachromosomal plasmid DNA, or due to integration of the entire plasmid, for example by homologous recombination at the P97 promoter sequence site (Figure 3B).

### Analysis of transposon insertion sites

**Figure 4:**
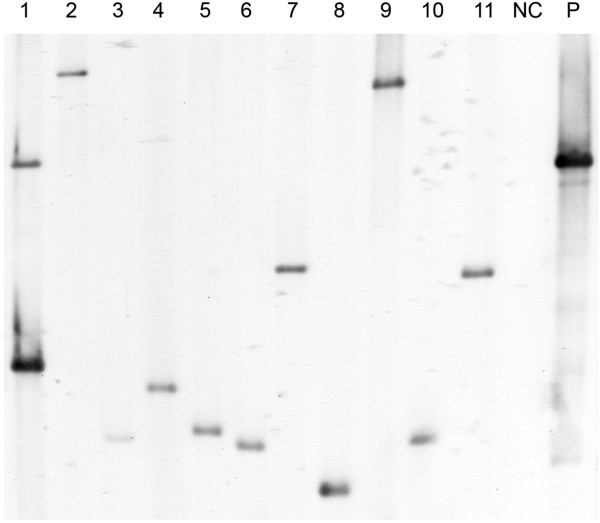
**Southern analysis of mutant *M. hyopneumoniae.*** Total DNA was extracted from 11 individual transformants of *M. hyopneumoniae* 232 generated using plasmid pMHC9-1, and subjected to Southern analysis using a probe specific for *tetM*. Single bands were generated for 10 of the mutants suggesting single transposon insertion sites. Two separate bands were present for mutant 1. No band was present for the untransformed *M. hyopneumoniae* strain 232 negative control (NC) and an intense band was present for the pMHC9-1 positive control (PC) as expected.

The presence of *tetM* and lack of plasmid backbone suggested that transposition of the transposon into the host chromosome had occurred. To locate the site of transposition in randomly selected transformants, a linker PCR technique was employed. Eleven individual tetracycline resistant colonies were colony-purified on Friis agar and cultured in Friis medium containing 0.5 ug/mL tetracycline. PCR was used to amplify across the site of insertion from the *tetM* to the linker. This was separately performed on DNA extracts using primer pairs to recognise both ends of the transposon. Analysis showed that a single dominant band was amplified for each transformant using one of the oligonucleotide primer pairs, consistent with integration of the transposon into the host chromosome at one site only. Occasionally, larger feint bands were also present, most likely due to particular PCR parameters such as long extension times and a high number of cycles, or by incomplete digestion of chromosomal DNA by the restriction enzyme *Alu*I. For several transformants, no PCR product was obtained with one of the two oligonucleotide primer pairs, but in all cases, where one primer pair failed, a PCR product was generated with the other primer pair. Therefore, it was possible to confirm independent transposon insertions for all of the 11 transformants. Additionally, the stability of transposon insertion sites following multiple serial passages of individual transformants in non-selective Friis medium was determined. Following a total of 15 passages in Friis medium containing no tetracycline all individual transformants retained their resistance to tetracycline upon subsequent culture in medium containing tetracycline. Linker PCR was repeated for each individual transformant, and agarose gel electrophoresis showed that the amplicons generated were identical in size to those obtained prior to serial passaging. Direct DNA sequencing of PCR amplicons was performed and this confirmed that the transposon had inserted into the host cell chromosome between thymine and adenine residues as expected. Where sequencing at both ends of the transposon was possible, the transposon insertion sites were found to match. Southern analysis was performed on total DNA extracted from the same 11 transformants to confirm that only single transposon insertions were present. For 10 of the mutants, a single band was present using a DNA probe specific for *tetM* contained within the transposon (Figure 4). For mutant 1, a small intense band was present along with a larger feint band. Only one insertion site was identified for this transformant by linker PCR and this insertion site was confirmed by DNA sequencing. It is possible that two bands were generated by Southern analysis by a failure to adequately digest the DNA to completion with *Hin*dIII.

### Insertion sites of transposon mutants

**Figure 5:**
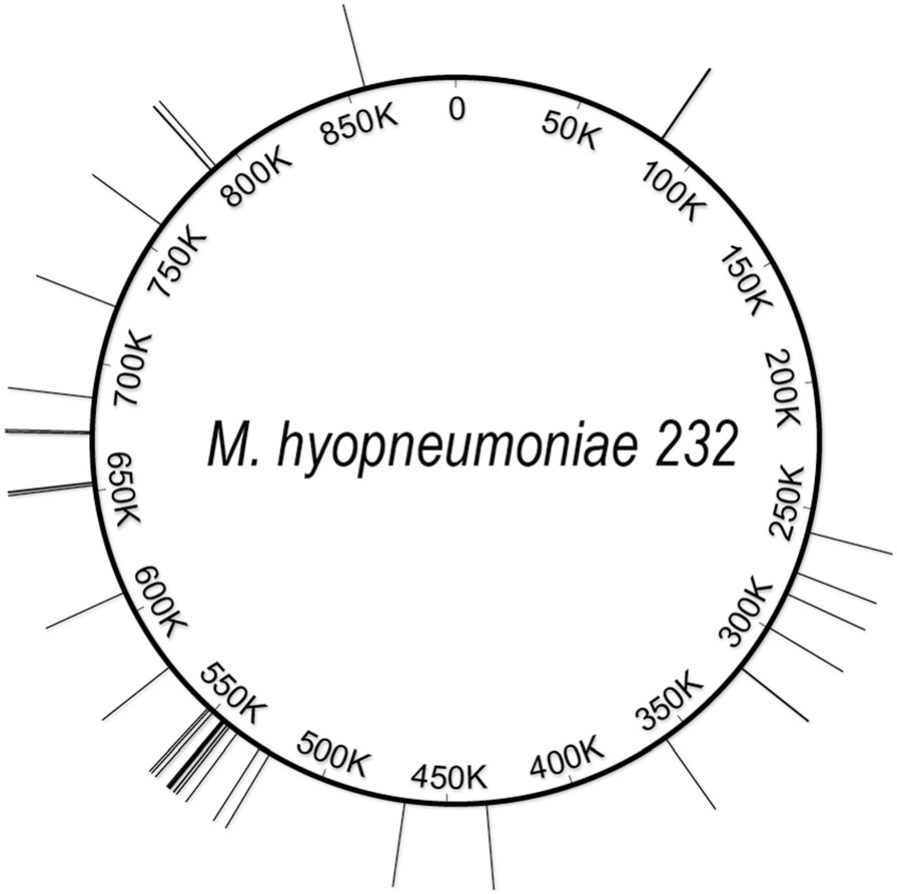
**Mapping of transposon insertions sites to the *M. hyopneumoniae *genome.** Transposon insertion sites were determined by linker PCR and sequencing of PCR products for 44 individual mutants. The location of these insertion sites in the *M. hyopneumoniae* strain 232 genome is depicted by lines emanating from the circular genome (drawn to scale). Thicker lines indicate the presence of more than one insertion at a given location.

Insertion site sequencing of a further 33 individual transformants was performed so that the locations of a total of 44 transformants were mapped to the *M. hyopneumoniae* strain 232 genome (Figure 5). Of these 44 transformants, 41 contained unique insertion sites (Table 3). Mutants 49, 68 and 161 all contained an insertion at the nucleotide 549968 in the *M. hyopneumoniae* strain 232 genome and mutants 56 and 66 contained identical insertion sites at nucleotide 574428. It was not possible to determine whether these mutants arose from a single transposition event or from multiple events at the same location. Following electroporation, mycoplasmas are given 3 hours to recover and express *tetM* in Friis broth without tetracycline prior to plating onto Friis agar containing tetracycline. It is possible that a single transposition event was able to undergo several rounds of multiplication during this period. In total, transposon insertions were present in 22 open reading frames (ORFs) and 5 non-coding regions (NCRs). For 5 ORFs, there was more than one transformant containing an insertion. The average size of an ORF in the *M. hyopneumoniae* genome is 388 amino acids (aa). All 5 of these ORFs were larger than 388 aa and it is therefore possible that more than one transformant was generated due to the larger size of these ORFs. However, a total of 11 transformants contained a transposon insertion in mhp447. Although this ORF is particularly large at 3970 aa, representing 1.3% of the *M. hyopneumoniae* strain 232 genome, it is likely that this represents a “hotspot” for transposon insertions. Similarly, 4 transformants contained insertions in the smaller *pepF* gene (608 aa) and this too may be over-represented. Most of the transposon insertions are in genes of unknown function but that appear to be dispensable for axenic growth, or in NCRs. Of the insertions in genes of known function, three were in the P97 ciliary adhesin and the P95 outer membrane protein. Insertions were found in 3 genes encoding phospotransferase system subunit proteins specific for fructose and ascorbate and in the molecular chaperone Trigger Factor. Several insertions were also identified in the *pepF* gene encoding oligoendopeptidase F and the *pepP* gene encoding a proline aminopeptidase. These are two of a small number of proteases that may be associated with processing of *M. hyopneumoniae* cell surface proteins [33].

**Table 3:** **Transposon insertion sites of *M. hyopneumoniae *** **strain 232 mutants.**

**Mutant**	**Transposon insertion location**	**ORF disrupted**	**Gene function**
72	84920	Mhp069	Unknown
11	85345	Mhp069	Unknown
40	259306	Tig	Trigger factor
159	275721	NCR	Unknown
50B	284811	Mhp261	Unknown
4	299711	Mhp271	P97 ciliary adhesion
96	318725	P95	Outer membrane protein
165	318913	P95	Outer membrane protein
91	359210	Mhp313	Unknown
9	434381	Mhp366	Unknown
34	466299	ulaA	PTS system ascorbate-specific transporter subunit IIC
37	522533	Mhp443	Unknown
54B	527019	Mhp445	Unknown
156	537514	Mhp446	Unknown
75	540819	Mhp447	Unknown
53B	541339	Mhp447	Unknown
102	542436	Mhp447	Unknown
90	542541	Mhp447	Unknown
1	544071	Mhp447	Unknown
78	544309	Mhp447	Unknown
24	544767	Mhp447	Unknown
5	545264	Mhp447	Unknown
68	549968	Mhp447	Unknown
49	549968	Mhp447	Unknown
161	549968	Mhp447	Unknown
52B	551617	NCR	Unknown
13	552503	NCR	Unknown
56	574428	NCR	Unknown
66	574428	NCR	Unknown
17	608509	Mhp490	PTS system fructose-specific transporter subunit IIABC
16	652232	pepF	Oligoendopeptidase F
162	653224	pepF	Oligoendopeptidase F
101	653333	pepF	Oligoendopeptidase F
92	653430	pepF	Oligoendopeptidase F
47	672237	Mhp535	Unknown
170	672318	Mhp536	Unknown
63	672979	Mhp537	Unknown
50	686279	Mhp542	Unknown
19	722926	ulaA	PTS system ascorbate-specific transporter subunit IIC
2	759509	NCR	Regulatory region of glpD
10	788260	Mhp630	Unknown
7	788457	Mhp630	Unknown
12	790862	Mhp631	Unknown
51B	856887	pepP	xaa-pro aminopeptidase

The locations of transposon insertions within the *M. hyopneumoniae* strain 232 genome for 44 individual pMHC9-1 transformants are shown. The open reading frame (ORF) or non-coding region (NCR) containing the insertion is shown and, where known, the function of the protein encoded.

### Generation of a large pool of *M. hyopneumoniae* mutants

We sought to determine whether our pMHC9-1 transposon delivery vector could be used to produce a large pool of random mutants, sufficient to provide an adequate number of “hits” in all of the non-essential genes of *M. hyopneumoniae*. A pool of at least 10 000 individual mutants would be expected to give a very high probability of an insertion into every one of the 692 protein coding sequences in the *M. hyopneumoniae* 232 genome determined by Minion et al. [27]. It was calculated that a pool of 10 000 individuals would, on average, contain a mycoplasma cell with an insertion every 89 bp, and that there would be 14.45 insertions per coding sequence. From the Poisson distribution, 10 000 independent insertions would give a 99.99995% probability of an insertion into each one of the 692 coding sequences. 20 individual transformations were performed using plasmid pMHC9-1 with approximately 10^6^ CFU per transformation. After 14 days growth at 37 °C on agar plates containing tetracycline, colonies were counted and harvested together in one large pool. A total of 11 759 colonies were counted across all 20 plates, representing an average transformation frequency of 3.4 × 10^-4^ transformants/CFU (standard error 5.3 × 10^-5^).

## Discussion

The inability to efficiently genetically manipulate *M. hyopneumoniae* has stood as a hurdle to advancements in the understanding of the pathogenesis of enzootic pneumonia in pigs. We recently reported the successful transformation of *M. hyopneumoniae* using an artificial plasmid system containing the origin of replication of *M. hyopneumoniae* and an antimicrobial resistance cassette, that was capable of self-replicating and maintenance in transformed bacteria [22]. We have used this system to optimise a number of conditions required for the successful transformation of *M. hyopneumoniae* by electroporation with plasmid DNA. This knowledge has now enabled the design and construction of a transposon-based system for generating random mutants in *M. hyopneumoniae*.

The Tn*4001* transposon is effective in generating insertional mutations in a number of different *Mycoplasma* species [16,18,19,34] but even using our optimised transformation conditions, we were unable to demonstrate transposition in *M. hyopneumoniae*. A Himar1 transposon delivery vector that was shown to be active in *M. gallisepticum* generated very low numbers of transformants in *M. hyopneumoniae* with a very restricted range of unique insertion sites [26]. By using an *M. hyopneumoniae* strain 232-specific promoter sequence to control the Himar1 expression and switching the wild-type Himar1 transposase for a hyperactive mutant C9 form, the frequency of transposition increased significantly. Analysis of transformants confirmed that resistance to tetracycline was due to a transposition event into the mycoplasma genome and it appeared that transposition occurred at a single site in each individual transformant. The transposon insertions appeared to be stable over serial passages and were maintained even in the absence of antimicrobial selection. These features are desirable when the phenotypes of mutants are studied in vitro, and particularly for in vivo studies of pools of mutants by TraDIS or signature-tagged mutagenesis. Additionally, we did not encounter any “pseudoresistant” colonies in our “no DNA” control transformations, which have been documented in transposon mutagenesis systems described for other mycoplasmas [16,26]. “Pseudoresistant” colonies can complicate the isolation of true transformants.

In the course of this study, it was evident that there were significant differences in the transformation frequencies obtained using plasmid pMHC9-1. We suspect that differences in transformation frequency can be partially accounted for by variations in the number of cells electroporated and/or the growth phase of the mycoplasma culture used. It appeared that an increase in the number of cells transformed did not necessarily result in an increase in transformation frequency. However, even where similar numbers of cells were electroporated, there could be significant differences in transformation frequencies, implying that other factors could be important. Growth of *M. hyopneumoniae* is assessed and monitored by a simple change in the phenol red pH indicator incorporated into the medium, owing to the fact that turbidity is not generated. Thus pH can serve as a rough guide for growth phase, but the actual number of cells can only be estimated after plating the culture out and allowing growth on agar medium for 5–7 days. Additionally, complex and undefined medium is used to grow *M. hyopneumoniae*, with 20% equine/porcine serum and freshly prepared yeast extract contributing to variations in batches of medium.

Transposon insertion sites were determined for a limited number of individual transformants using a linker PCR technique and direct sequencing of PCR products by Sanger sequencing. From our limited analysis, we found transposon insertions in a number of *M. hyopneumoniae* genes of both known and unknown function, and in non-coding regions. We observed a clustering of transposon insertions in mhp447, a large open-reading frame of unknown function. The Himar1 transposon inserts at 5’-TA-3’ dinucleotides and we considered that the large number of insertions may be due to an increase in AT content. However, analysis of this ORF showed that the AT content was not unusual at 0.681 compared to 0.714 for the entire *M. hyopneumoniae* strain 232 genome. Alternatively, it is possible that there are hotspots for Himar1 insertions in the *M. hyopneumoniae* strain 232 genome, that may be affected by factors such as DNA topology, as is the case with other transposons, including Mariner elements [35]. It is difficult to draw any conclusions from analysis of such a small pool of mutants, but determination of the insertion sites of a large pool of mutants by next generation sequencing would answer the question of whether favoured insertion sites do occur. Using plasmid pMHC9-1, we were able to produce a large pool of mutants. Over 10 000 colonies were generated through a number of transformations that, given an even distribution should be expected to have a high probability of insertions into all of the genes of *M. hyopneumoniae*. This pool of mutants may prove a valuable tool in future studies of *M. hyopneumoniae* in allowing a list of minimal essential genes to be determined. Such a pool of mutants could also be subjected to an in vivo screen for attenuated mutants using powerful functional genomic techniques such as TraDIS.

A novel transposon delivery system has been generated for *M. hyopneumoniae*, allowing for the first time the production of large numbers of random transposon insertion mutants. This system has been used to generate mutants with random transposon insertions in individual *M. hyopneumoniae* protein coding and non-coding sequences. It has also been used to generate a large pool of over 10 000 transposon insertions. These mutants are powerful tools in elucidating the functions of *M. hyopneumoniae* genes, and in turn unraveling the pathogenesis of this important swine pathogen.

## Competing interests

A patent application covering the method of transposon mutagenesis described in this paper has been filed by the Royal Veterinary College. The authors declare that they have no further competing interests.

## Authors’ contributions

Experiments were conceived by GAM, ANR and BSC. Experiments were performed by GAM and ASD. BSC, JTB, ANR, SEP, PRL, BWW, AWT and DJM assisted with experimental design. The manuscript was written by GAM and ANR and was approved by all authors.

## Supplementary Material

Additional file 1 BRaDP1T consortium. List of researchers collaborating in the BRaDP1T consortium. Click here for file
